# Molecular characterization of signalling pathways in cancer stem cells

**DOI:** 10.3332/ecancer.2008.115

**Published:** 2008-12-02

**Authors:** C Regenbrecht, Y Welte, A Jodicke, R Hugel, P Walden, M Jung, H Lehrach, J Adjaye

**Affiliations:** 1 MPI for Molecular Genetics, Berlin, Germany; 2 Clinical Research Group tumour Immunology, Department of Dermatology, Charite’-Universitatsmedizin, Berlin, Germany; 3 Clinic for Neurosurgery, Vivantes Klinikum Neukolin, Berlin, Germany

## Abstract

To avoid artefacts introduced by culturing cells for extended periods of time, it is crucial to use low-passage patient-derived tumour cells. The ability to enrich, isolate and assay sub-populations of cells that behave as cancer stem cells (CSCs) from these primary cell lines is essential before performing characterizations such as gene-expression profiling. We have isolated cells from glioblastomas which show characteristics of CSCs. Although glioblastomas contain only a relatively small amount of putative CSCs, these cells express many genes which seem to be worthy targets for future therapies.

## Introduction

Al-Hajj *et al* were the first to identify and prospectively isolate a minority sub-population of cells from human solid tumours that contained all of the ***in vivo*** tumour-forming abilities. The tumourigenic cell population was identified based on its cell surface phenotype. This population could initiate tumours in immuno-compromised mice with as few as 200 cells, while as many as 500,000 or more of the remaining cells in the tumour did not initiate new tumours in mice [1].

Fang *et al* have shown that upon culturing of metastatic melanoma cell suspensions under appropriate conditions, a subset of cells could be propagated as non-adherent spheres, which could then be induced to differentiate ***in vitro*** and to generate tumours ***in vivo*** [2]. The ability to acutely isolate and assay sub-populations of cells from tumours that behave as cancer stem cells (CSCs) is essential before performing characterizations such as gene-expression profiling, to avoid artefacts introduced by culturing cells for extended periods of time.

## Results

### Tissue samples

**Figure 1:**
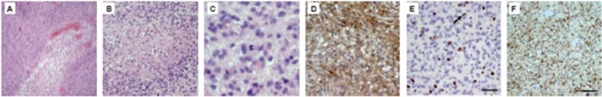
H&E staining of glioblastoma tissues shows typical histological features of the tumour (A–C). In (A), vessel proliferates are present; (B) shows an area of extensive necrosis, while (C) displays mitotic cells within the tumour. Immunohistochemical characterization of the removed tissue confirms the diagnosis. (D) shows staining against GFAP, a marker for glial origin. (E) KI-67 antibody is used to show the high mitotic activity in the tumour tissue. (F) Proof of neoplastic character of the tissue, using an anti-MAP2C antibody.

Glioblastoma (GBM) tissues were obtained intra-surgically. A trained pathologist evaluated stage and grading of the removed tumour tissue. For our experiments, we only cultivated cells from tissues containing at least 90% tumour cells as determined by H&E staining (Figure 1A–C). In a more elaborate diagnosis, we determined glial origin (GFAP) (Figure 1D), mitotic activity (KI-67) (Figure 1E) and neoplastic character (MAP2C) (Figure 1F).

### In vitro *generation of neurospheres*

**Figure 2:**
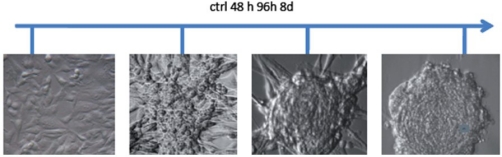
Phase contrast micrographs from human GBM cells treated with ES-medium + FGF2. Untreated cells cultivated in normal medium grew as monolayers. Under ES cell conditions, the cells began to change their morphology and developed a spherical, colony forming phenotype. After eight days of cultivation under these conditions the phenotype remained stable and the spheres began to detach from the substrate.

Tissues that met our criteria were used for low-passage cell culture. We enriched CSCs using embryonic stem (ES) cell medium supplemented with FGF2 [3]. Cells cultured under these conditions began to change their morphology towards a colony-forming phenotype (Figure 2). This phenotype was similar to that of hES cells cultured under the same conditions. Further analysis showed that the phenotype remained stable for at least four weeks and was reversible by FGF withdrawal.

To show the de-differentiation towards a more primitive neural phenotype, we performed immunofluorescence microscopy of cells from the normal medium and ES medium with NESTIN.

### Cell sorting

**Figure 4:**
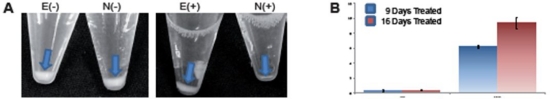
(A) shows the different yields of CD133– and CD133+ cells after MACS sorting. E(–): negative fraction from ES medium; N(–): negative fraction of cells cultured in normal medium; E(+): CD133+ fraction of cells cultured in ES medium; N(+) CD133+ cells isolated from cells grown in tumour medium. The yield of CD133+ cells increases upon duration of stimulation. (B) shows the quantification of CD133+ cells after nine and 16 days of stimulation in ES medium.

To investigate if the treated cells were enriched for putative cancer stem cells, we employed magnetic sorting with a bead-coupled anti-CD133 antibody. Enrichment of CSCs by selective cell culture methods resulted in higher yields of CD133+ cells (Figure 4A). This enrichment was time dependent, with its maximum yield after 16 days of stimulation (Figure 4B).

**Figure 5:**
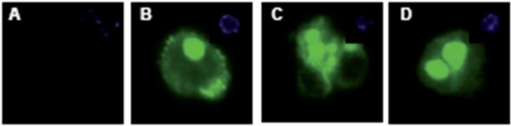
CD133+ and CD133– cells were stained for CD133 expression. The negative fraction (A) showed no CD133 expression, while the majority of cells from the CD133 positive fraction had strong expression of the protein (B–D). DAPI inset.

To determine the quality of enrichment, we kept the cells from the positive and negative fractions in culture for four days and stained them with a fluorescence coupled anti-CD133 antibody (Figure 5).

### Pathway analysis

**Figure 6:**
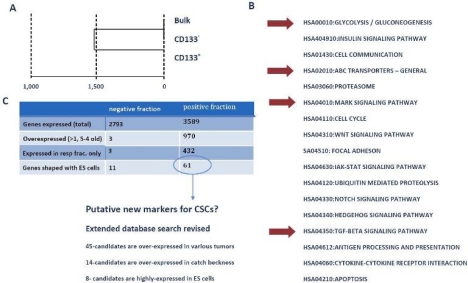
(A) Cluster analysis of expression profiling from GBM1207 bulk, CD133+ and CD133– cells. (B) GO- and KEGG analysis of CD133+ revealed a set of activated pathways, known to be important for oncogenesis. Although most tumours harbour only a small subset of CSCs, these cells might have a high impact on the properties of the tumour. Red arrows mark pathways, which came up in the analysis of CD133+ cells and hES cells. (C) Comparison of CD133+/– fraction with human ES cells. The negative fraction shares only 11 genes with hES cells, while 61 genes are expressed exclusively in CD133+ and ES cells. From these 61 candidates, 46 are known to play a role in various cancers and eight targets are most commonly expressed in hES cells. These 61 genes could provide the basis for the identification of new CSC markers and future drugs.

**Figure 7:**
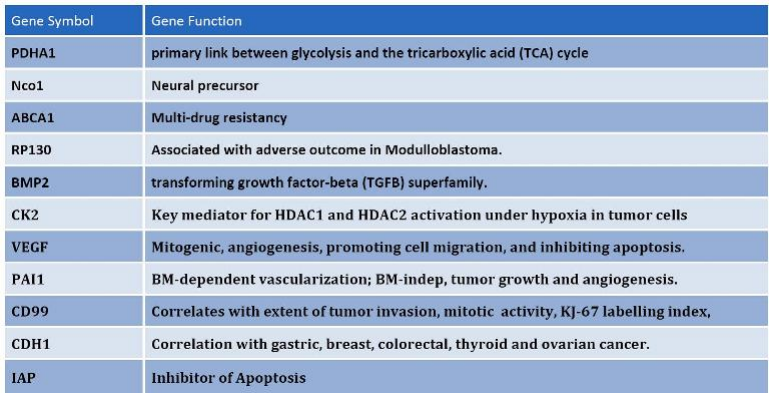
Among the differentially expressed genes that are shared between CD133+ and hES cells, common regulators of oncogenisis and markers for prognosis were identified. These proteins cover a variety of properties, which include angiogenesis, inhibition of apoptosis, multi-drug resistance and cell-cycle control proteins.

To identify the mechanisms and pathways underlying CSCs, we conducted RNA-expression analysis using the Illumina platform. For expression analysis, we used CD133+, CD133– and bulk cells from the GBM1207 cell line and compared this data to the expression profile of hES lines H1/H9 (Figure 6). Expression analysis revealed a group of 11 genes that were over-expressed and known to play a role in the progression of various malignancies (Figure 7).

**Figure 8:**
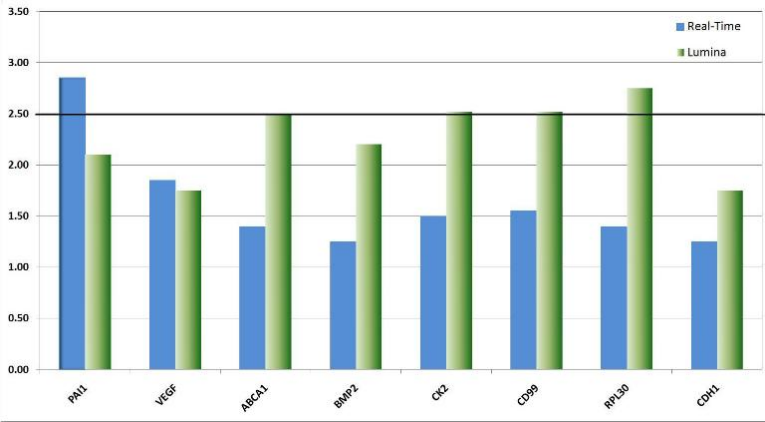
Real-time validation of eight candidate genes identified by genome-wide expression profiling. The relative over-expression of these genes indicates that CSCs despite their rare distribution in the tumour tissue might have a strong influence on the descended cells. The over-expression of genes like VEGF point to the fundamental role CSCs might have on the establishment and maintenance of a tumour promoting micro-environment or niche. Other genes like CK2 function as interfaces in a plethora of cancer relevant pathways such as p53, wnt-signalling and Ç-catenin.

We validated the differential expression of eight selected marker genes from the expression analysis by real-time PCR (Figure 8). All markers were found significantly (>1.5-fold) over-expressed in CD133+ cells compared to the respective negative fraction.

## Conclusion

Still only little is understood about the origin and nature of CSCs [4]. From our findings, we conclude that although GBMs contain only a relatively small amount of putative CSCs, these cells express many genes, which seem to be worthy targets for future therapies. Among these genes, we found important signalling molecules for angiogenesis, apoptosis, multi-drug resistance and metastasis. A large number of markers that have clinical relevance for the evaluation of prognosis in various tumours show increased expression in CD133+ cells compared to their CD133– counterparts. Additionally, these CD133+ cells share important pathways with human ES cells, which help explaining self-renewal capacity, drug-resistance and other properties credited to stem cells and cancer.

## Figures and Tables

**Figure 3:**
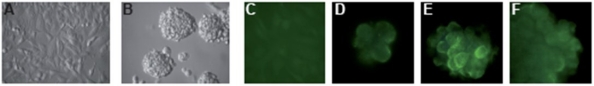
We stained both, adherent monolayer (A) and colony (B) grown cells against Nestin. Nestin is a type VI intermediate filament, which is commonly used as a marker for neuronal precursor cells. Untreated cells do not express Nestin (C), while it was expressed in all spheres (D–F).
